# Contactless mass transfer for intra-droplet extraction

**DOI:** 10.1038/s41598-020-64520-4

**Published:** 2020-05-06

**Authors:** Shusaku Asano, Yu Takahashi, Taisuke Maki, Yosuke Muranaka, Nikolay Cherkasov, Kazuhiro Mae

**Affiliations:** 1 Institute for Materials Chemistry and Engineering, Kyushu University, 6-1, Kasuga Koen, Kasuga, 816-8580 Japan; 2 Interdisciplinary Graduate School of Engineering Sciences, Kyushu University, 6-1, Kasuga Koen, Kasuga, 816-8580 Japan; 3 Department of Chemical Engineering, Graduate School of Engineering, Kyoto University, Kyoto-daigaku Katsura, Nishikyo-ku, Kyoto, 615-8510 Japan; 4 School of Engineering, University of Warwick, Coventry, CV4 7AL United Kingdom

**Keywords:** Chemical engineering, Process chemistry

## Abstract

This study demonstrates the possibility of “contactless” mass transfer between two aqueous slugs (droplets) separated by an oil slug in Taylor flow inside milli-channels. Separation of the alternating aqueous slugs at the outlet was performed by switching a couple of solenoid valves at branched outlets according to signals obtained by an optical sensor at the branch. Transfer of bromothymol blue (BTB) from acidic to basic aqueous slugs was performed for demonstration. In some cases, aqueous slugs separated by oil, merged catching on each other due to the velocity difference. Interfacial tension which was affected by the solute concentration was responsible for the velocity difference. Position-specific mass transfer activity at the rear end of the aqueous slugs was found on the course of the experiment. A meandering channel decreased the velocity difference and enhanced mass transfer. Almost complete (93%) transfer of BTB was achieved within a short residence time of several minutes under optimized conditions. The presented system opens a way for advanced separation using minimum amounts of the oil phase and allows concentrating the solute by altering relative lengths of the sender and receiver slugs.

## Introduction

Slug flow^1^ (also referred to as segmented^2^ or Taylor flow^3^) is one of the most attractive flow patterns for immiscible fluids because of exceptionally high mass transfer and separation of individual slugs from each other^4^. A fluid with higher wettability to the wall becomes a continuous phase, and the other fluid forms dispersed slugs^5^.

Rapid mass transport between different phases in the slug flow is enabled by a large surface-to-volume ratio and internal circulation^6^. Mass transport rate in the slug flow can be orders of magnitude faster than conventional batch mixing processes^7^. Such rapid transport enhances liquid-liquid extraction^8^, gas absorption^9^, and heterogeneous reactions^10^. In a field of lab-on-a-chip, compartmented slugs are now utilized as a screening platform with slugs used as independent reactors^11^. Reaction conditions can be optimized by changing concentrations individually inside slugs. When set concentrations are different in the slugs, mass transport between the slugs is undesired because it changes concentrations and contaminates the nearby slugs^12^. Inert and immiscible phases such as fluorinated oils are preferred as a continuous phase for preventing such undesired intra-droplet mass transport^13,14^.

In this work, we conceived the idea to use this undesired intra-droplet mass transport to move materials in between the spatially separated slugs. With the sequential mass transport from one aqueous slug to the continuous oil phase, and then to another aqueous slug, we can dramatically increase the potential and possibility of slug flow for applications in extraction and reaction. The advantages are numerous. First, such operation eliminates back-extraction (which usually requires post-processing after extraction)^15,16^ and creates a single unit operation process. Second, the consumption of extractant chemicals can be dramatically reduced. For the advanced extraction such as radioactive materials^17^ and chiral molecules^18^, expensive extractants are specially designed for selectivity. By releasing the molecule to another aqueous phase continuously, extractant could maintain its extraction ability with minute amounts. Third, the reactive extraction process can be designed more flexibly. Previous studies on chemical reaction have utilized slug flow for supplying poorly soluble reactant to the reaction phase^19^, efficient use of the phase transfer catalyst^6^, and extracting the product from the reacting phase for preventing overreaction^20^. Adding one more phase to these conventional slug flow process opens applications in (i) supplying reactants from one slug to another slug at a controlled rate, and (ii) extracting products from one slug and further converting in another slug.

Demonstration of the application of the intra-slug mass transfer also requires a method for separating and sorting individual slugs from a stream. Without sorting the slugs, all the aqueous slugs will be mixed at the outlet. Such droplet sorting at the outlet had been noted a decade ago^21^. However, there are few reports that practically demonstrate droplet sorting to designated outlets^22^. In this study, we show the concept of intra-slug material transfer and a simple system for the detection and sorting of slugs at the milli-reactor outlet.

## Results

### System for slug formation, detection, and sorting

**Figure 1:**
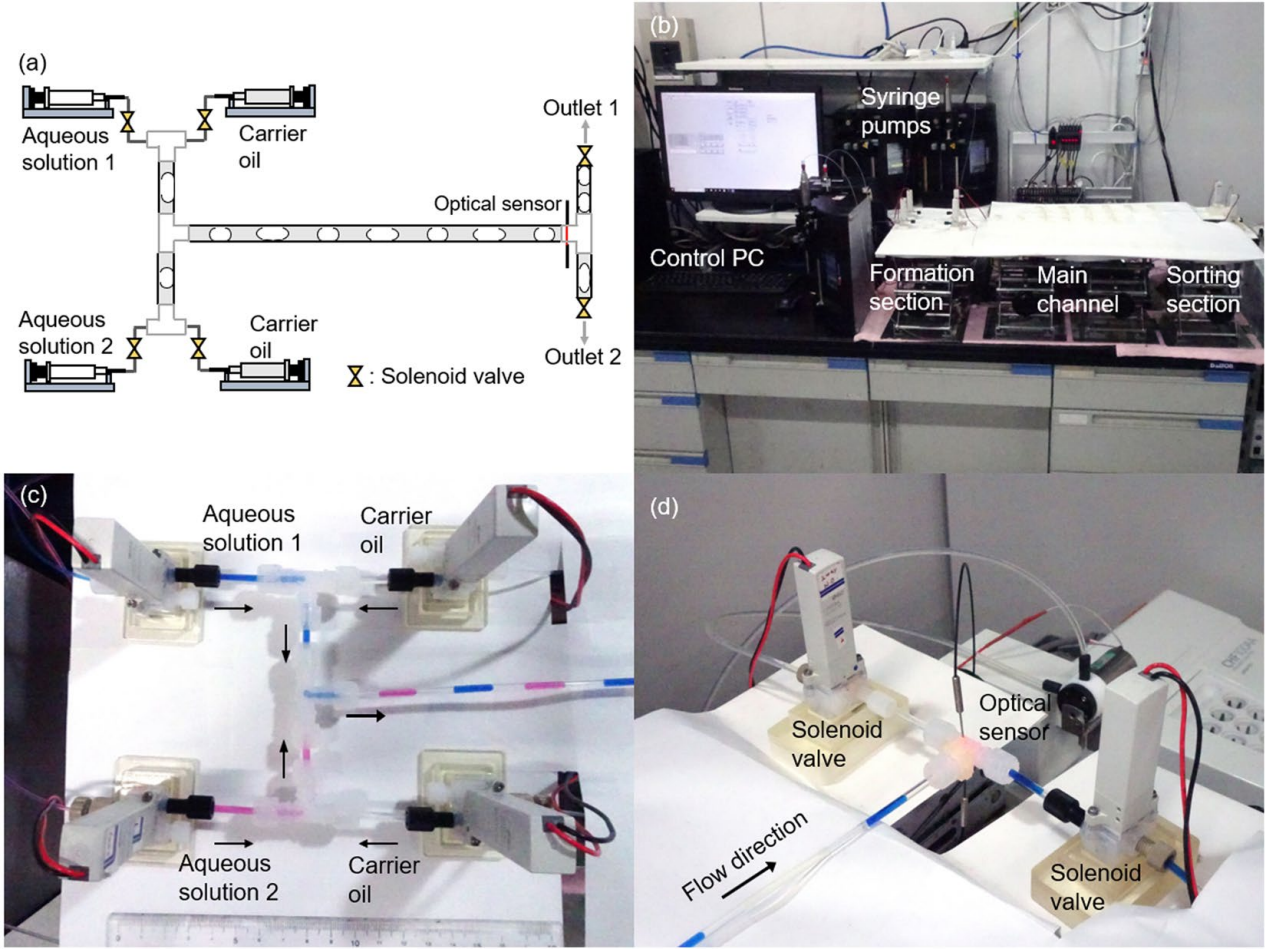
A system used to form water-oil slugs of two compositions and their separation (sorting): (**a**,**b**) overview of the system, (**c**) slug formation section with 4 solenoid valves, (**d**) slug sorting section with 2 solenoid valves and a tee equipped with an optical sensor.

Figure 1 shows the experimental setup used to generate and sort a sequence of oil-water slugs of two compositions. The setup contains 4 syringe pumps, 6 solenoid valves, and an optical sensor, all controlled by a computer with a bespoke LabVIEW software. Four inlet streams (two different aqueous phases and carrier oil) are combined into tees using the solenoid valves (Fig. 1a,c).

We studied two different pump models and three operation modes where the flows of syringe pumps are controlled by the solenoid valves, the syringe pumps, or both. The results described in the Supplementary SI1 and Supporting Movie 1 show that the combined control using the pumps and the valves generated consistent aqueous slugs.

**Figure 2:**
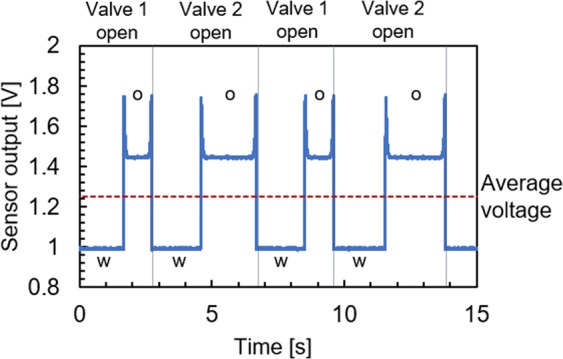
The output of the optical sensor attached to the sorting tee for the colorless aqueous slugs (pure water, “w”), and a colorless carrier oil (dodecane, “o”) at the total flow rate of 2.0 mL min^−1^, and set aqueous slug length of 15 mm.

Once the aqueous slugs were generated and passed through the reactor, they were sorted using a system of solenoid valves controlled by an optical sensor placed inside the outlet tee joint. The sensor contained an optical fiber opposite to a 630 nm light-emitting diode (LED), discussed further in Supplementary (SI2). The sensor output shown in Fig. 2 demonstrates the versatility of the approach for a broad range of liquids. The figure shows the possibility of distinguishing between even the colorless aqueous and oil phases based on various refractive indexes^23^.

### Intra-slug mass transfer demonstration

**Figure 3:**
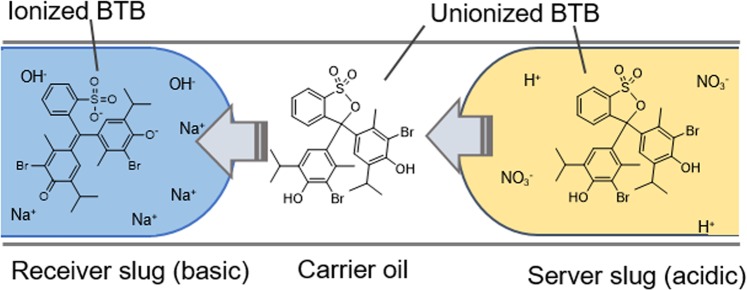
Schematics of the BTB transportation.

The sensor was shown to be efficient in separating optically transparent media. For the sake of visualization, however, we examined mass transfer between slugs using bromothymol blue (BTB) dye as the transferred molecule, shown in Fig. 3. An acidic solution (0.5 M HNO_3_) of BTB was used as a sender slug. The BTB molecule is unionized in acid and is easily extracted by a non-polar oil (dodecane). Afterward, BTB is further extracted by the receiver slugs and becomes deprotonated in the aqueous basic solution (0.5 M NaOH). The mass transfer process can be visually examined because of the yellow color of the unionized BTB molecule and the blue color of the deprotonated BTB molecule.

**Figure 4:**
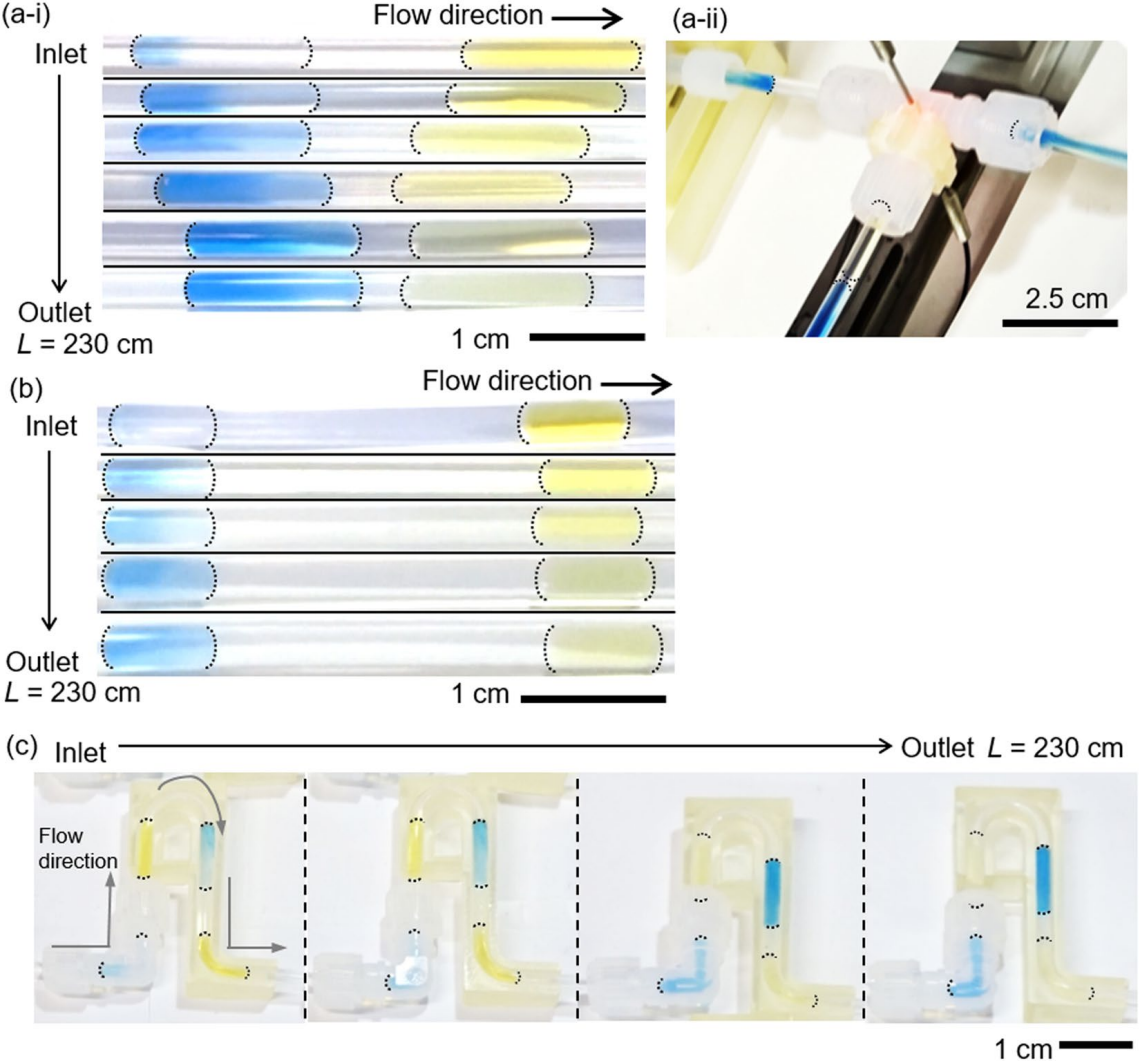
Photographs of BTB transfer experiment: (**a**) run 1, straight channel and 50 vol% aqueous flow (i) color and distance change and (ii) slug merging at the outlet sorting tee, (**b**) run 2, straight channel and 23 vol% aqueous flow, (**c**) run 3, meandering channel and 50 vol% aqueous flow. The total flow rate was 10 mL/min, and the channel volume – 9.0 mL. Aqueous slugs were 15 mm long for (**a**,**c**); 7.5 mm long for (**b**). End caps of the slugs are highlighted for clarity.

Figure 4a and Supporting Movie 2 show the experiment (run 1 in Table 1) with a straight channel made of PFA tube with an internal diameter (ID) of 2.18 mm and 2.4 m length. The mass transport through the oil phase was confirmed by the disappearance of yellow color in the sender slugs and appearance of blue color in the receiver slugs. However, two problems were observed in this experiment. Firstly, mass transfer in the straight tube was limited as indicated by the colorless front sections of the receiver slugs. The colorless part of aqueous slugs shows that there is a limited internal recirculation inside the aqueous receiver slugs. Secondly, the receiver slugs moved faster than the sender slugs. Distance between the slugs decreased as they moved along the channel. As a result, a slug merging occurred at the sorting section as shown in Fig. 4a.

**Table 1:** Bromothymol blue (BTB) transfer from an aqueous sender slug through an oil into a receiver slug at the total flow of 10 mL min^−1^.

Run No.	*V*_channel_ [mL]	*L*_channel_ [m]	Channel type	*L*_sender_ [mm]	*L*_receiver_ [mm]	*ϕ*_H2O_ [%]	*X*_sent_ [%]	*X*_receive_ [%]
1	9.0	2.3	straight	15	15	50	Slugs merged	
2	9.0	2.3	straight	7.5	7.5	23	82	33
3	9.0	2.3	meandering	15	15	50	88	63
4	9.0	2.3	meandering	45	15	67	79	53
5	13.7	3.2	meandering	30	15	60	91	74
6	25.4	6.0	meandering	30	15	60	97	85
7	29.7	7.1	meandering	30	15	60	98	93

*L* – lengths of the corresponding slugs; *ϕ*_H2O_ – volume fraction of aqueous phase in the flow; *X*_sent_, *X*_receive_ – a fraction of the initial BTB amount lost by the sender and obtained by the receiver slugs.

To avoid aqueous slugs merging, the length of oil slugs was increased (aqueous flow fraction decreased to 23 vol%) as shown in Fig. 4b and Supporting Movie 3. The nominal lengths of the sender and receiver slugs decreased to 7.5 mm. Under these conditions, the velocity difference between the consecutive slugs decreased. The slugs were separated well at the sorting tee and merging avoided. However, the mass transfer rate also decreased significantly. Blue color intensity of the receiver slugs was much lower compared to run 1.

A combination of meandering 3D-printed channels and flow path constrictions were introduced to intensify mixing inside the slugs. Meandering channels is a simple way to create circulation due to the difference in the inner and outer channel wall lengths^24–26^. The constrictions/expansions of the flow path created with PFA tubes having different ID further enhanced circulation by squeezing and expanding the slugs. Figure 4c and Supporting Movie 4 show the results where both the stagnant colorless zones and slug merging were eliminated.

BTB concentrations after the channels were measured with the ultraviolet-visible (UV-Vis) spectroscopy (Table 1). With the straight channel and short aqueous slugs (7.5 mm long, run 2), the sender slugs lost 82% of initial BTB and the receiver slugs collected 33%, the rest remained in the oil phase. With the meandering channel and 15 mm slugs, about twice as much BTB was transferred to the receiver slug (63%, run 3). The experiments with the larger sender slugs were conducted to examine the possibility to make the BTB concentration in the outlet receiver slugs larger than that of inlet sender slugs. When the sender slugs were 3 fold longer than the received slugs (run 4), 53% BTB was successfully transferred resulting in a 1.6-fold increase in BTB concentration. To complete the mass transfer process, experiments with increasing the tubing lengths (residence time) were conducted using 13.7 mL (run 5), 25.4 mL (run 6) and 29.7 mL (run 7) channels. BTB concentration increased with the increase in channel length. Almost complete transfer (93%) was confirmed in a 30 mL channel with the residence time of only about 3 min.

### Slug velocity measurement

The experiments showed that slug merging occurs under some operating conditions indicating that the sender slugs have a lower velocity compared to the receiver slugs inside the channel. Hence, the slug velocity measurements were conducted for varying slug components to clarify the mechanism (Table 2). Here, velocity excess is defined as an increase in the observed slug velocity relative to the calculated superficial velocity; ${(u}_{{\rm{s}}}-{u}_{{\rm{PFR}}})/{u}_{{\rm{PFR}}}$, where *u*_s_ is the measured slug velocity and *u*_PFR_ is the superficial velocity calculated from the total flow rate and the area of channel cross-section. The slug velocity was measured by the video analysis of the moving slugs with the standard deviation below 0.2%.

**Table 2:** Effect of the slug composition on the velocity.

Entry	Channel	Oil phase	Aq. slug 1	Aq. slug 2	*V*_1_ [%]	*V*_2_ [%]
1	straight	dodecane	water		0.6	0.7
2			receiver solution		1.0	1.2
3			sender solution without BTB		1.2	1.1
4			sender solution		2.4	2.3
5			sender solution	receiver solution	1.5	2.0
6			receiver solution with 0.2 mM BTB		3.2	3.2
7		dodecane with 0.1 mM BTB	receiver solution		2.3	2.3
8	meandering	dodecane	receiver solution		2.6	2.6
9			sender solution		3.2	3.1
10			sender solution	receiver solution	2.5	2.9
11			receiver solution with 0.2 mM BTB		3.6	3.5

*V*_1_, *V*_2_ are excess velocities for aqueous slugs 1 and 2. Component effect on the slug velocity at the total flow of 10 mL min^−1^, slug length 15 mm, and aqueous fraction 50%. Sender solution: 0.5 M HNO_3_, 8wt % ethanol, 0.2 mM BTB; receiver solution: 0.5 M NaOH, 8wt % ethanol. The same aqueous slugs were introduced using the solenoid valve system to ensure consistency with the mass transfer experiments, except for entry 5 and 10.

In liquid-liquid slug flow, the continuous phase creates a film between the dispersed slug and the channel wall^27,28^. This film decreases (i) the effective channel diameter for the dispersed phase, and (ii) the friction with the wall (lubrication effect) – both effects result in the increasing droplet velocity^13^. Film thickness, as well as its lubrication effect, are influenced by the capillary number (Ca)^29–31^, the ratio of the viscous drag and the surface tension forces.

Ferraro *et al*. reported that adding ethanol to aqueous slugs decreases the interfacial tension and increases the slug velocity^13^. Our results agree – the slug velocity increased for the receiver solution slugs (8 wt% ethanol, 0.5 M NaOH) compared to pure water slugs (Table 2, entries 1, 2). Acid and base in the sender or receiver slugs did not play an important role – the same velocity was observed for the sender solution slugs without BTB (entry 3). With BTB, the velocity excess of the sender solution increased by 1.2% (entry 4). We showed that BTB works as a surfactant decreasing the interfacial tension between aqueous droplets and carrier oil from 33.57 mN m^-1^ to 20.84 mN m^-1^ with the addition of 0.2 mM BTB (Supplementary, SI3**)**. Note that the influence of impurities on the surface property of the droplet is well known and not specific to BTB in this study^31,32^. In the alternating slug system of sender and receiver slugs, the velocity excess of sender slugs decreased to 1.5% and that of receiver slugs increased to 2.0% (entry 5). Surface tension change accompanying with BTB transfer reasonably explains the tendency. With the removal of BTB from sender slugs, the velocity of the sender slugs is expected to decrease to that of the sender slugs without BTB. At the same time, BTB extraction into the receiver slugs increases their velocity.

The observed velocity change with BTB transfer also explains the reason why the receiver slugs in run 2 (Table 1) were not catching up with the sender slugs. Due to the slow mass transfer in run 2, the acceleration effect by BTB would not be sufficient for receiver slugs to catch up with the sender slugs. Note that all the velocity measurements were conducted in a center part of the 1 m tubing. Different positions would result in different results owing to BTB transfer. The velocity increase of receiver slugs in the presence of BTB in the oil phase was separately confirmed by adding BTB into receiver slugs or dodecane (entry 7). The acceleration effect was the largest for the case of BTB in the receiver slugs (entry 6).

Replacing straight with meandering channels increased the velocities of all the droplets likely due to a thicker oil film and deformed slug shape. The dispersed phase cannot reach the edge of the bending section so that the continuous phase forms a thicker film^33,34^. The curved channel would also enhance the inter slug convection, which is important for deforming the slug and increasing the slug velocity^35^. This velocity increase was the highest for a pair of receiver solution slugs which did not contain BTB (entries 8, 2). For a pair of sender slugs, the velocity increase compared to the straight channel was 0.8% (entries 9, 4), about half of the pair of receiver solution slugs. Importantly, the velocity difference between the sender and receiver slugs decreased in the meandering channel by 0.1% (entry 10, 5) decreasing the probability of slug merging. The velocity increase by channel modification has relatively reduced the impact of the velocity increase by the surface tension difference.

### Mechanistic investigation on the BTB recovery

**Figure 5:**
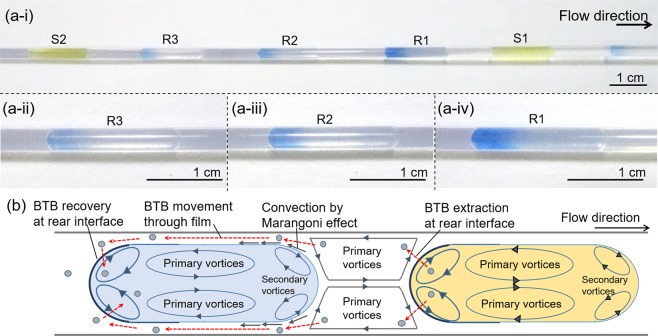
BTB transfer mechanism: (**a**) experimental evaluation with 3 receiver slugs between 2 sender slugs; (i) entire configuration and, (ii)-(iv) closed-up images of receiver slugs taken at the fixed observation point of 70 cm from the inlet tee (total flow rate 10 mL min^−1^, aqueous slugs length 15 mm, 50% aqueous fraction). (**b**) proposed scheme of “rear-to-rear” mass transfer.

During the mass transfer experiments, the color of the receiver slugs changed only at the tail section. To investigate the phenomenon, mass transfer visualization was conducted with a series of three receiver slugs surrounded by two sender slugs shown in Fig. 5a and Supporting Movie 5. Receiver slugs are named as R1, R2, and R3 according to the order of injection. The position of the color change was still the tale part for all the slugs. Interestingly, the blue color intensity observed at a fixed position in the channel was the highest for R1 and the lowest for R3. The order of color intensity strongly suggests that BTB was transferred from the rear of the sender slugs to the rear of the receiver slugs; not from the front of the sender slugs to the rear of the receiver slugs (Fig. 5b).

 The proposed transportation route of BTB from the sender slug to the receiver slug is illustrated as the dotted arrow in Fig. 5b. Extracted BTB from the sender slugs is carried by the primary vortices of the carrier oil to the front part of the receiver slugs. Then BTB moves to the rear end of the receiver slugs through the oil film between the receiver slugs and the tubing wall and finally extracted to the receiver slugs with help of rear secondary vortices. Secondary vortices in the front and rear section of the slugs were observed in the literature^36–38^. Primary vortices circulate in the central section of the slugs; the secondary vortices circulate only in the cap regions. The occurrence of the secondary vortices is determined by the capillary number (Ca) and the viscosity ratio of continuous phase to dispersed phase (*λ*), which were 0.00321 and 0.765 – the values that indicate the formation of the secondary vortices^39^. Detailed physical properties of each phase are listed in the Supplementary SI3, 5. Blue color stagnation at the rear of receiver slugs (Fig. 5a) also supports the existence of the secondary vortices which do not enter the center part of the slug. Hence, the secondary vortices at the rear part contribute to the interfacial mass transportation.

 The oil film thickness estimated by the Bretherton’s equation^29,30^ is 31.8 μm (Supplementary, SI5). The Fourier number Fo, which shows the ratio between residence time of slugs on the film and the diffusion time from film to slug, was 0.11. Such a low value (Fo < 1) supports the assumption that BTB transfer was low through the film diffusion. The thickness value seems too low to carry enough BTB from the front section to the rear section by convective motion. Considering the surfactant properties of BTB discussed in the previous section, the Marangoni effects may play an important role in promoting the BTB movement through the film. The gradient of the surface density of the surfactant generates a Marangoni stress which causes the liquid to flow from regions from low surface tension to high surface tension^32^.

## Discussion

We successfully demonstrated the concept of contactless mass transfer utilizing alternating slugs. The mass transfer was completed in about 3 min and outlet concentration of the receiver slugs was larger than that of sender slug inlet. An increase in the concentration clearly shows that mass transfer is not due to the physical mixing of two aqueous droplets. BTB transfer through the carrier oil was driven by the stability difference for BTB in two aqueous droplets.

The most critical issue in the operation of contactless mass transfer was the velocity difference of the slugs induced by the difference in surface tensions. A higher velocity of the receiver slugs may result in them catching up the sender slugs and merging before sorting. There are two approaches to avoid slug merging. One is a passive approach that designs the channel dimensions and operating conditions by predicting the slug velocity. The other is an active approach that dynamically adjusts the slug distance during the operation. Ferraro *et al*. proposed and demonstrated the passive approach for controlling the droplet distance by adjusting the surface tension of each droplet^13^. However, in the case of mass transfer applications, the passive approach with designing surface tension is not applicable because the surface tension changes during the operation. The data show that even 0.2 mM of BTB transfer create a significant velocity difference. Hence, the active approach is practicable for expanding the applications of the contactless mass transfer system. One possibility is the additional injection of carrier oil in the middle of the channel. With the combination of optical sensors and a syringe pump, selective injection of oil is possible to bring the shortened slug distance back to the initial distance. The active injection system is under construction in our group and will be used in future studies.

In the course of the experiment, we confirmed the position-specific mass transfer of BTB at the rear cap region. There are many studies focusing on the mass transfer in slug flow^9,39–42^. However, to the best of our knowledge, none of them remarked the excelling activity of the rear cap nor the inert activity of the front cap. One possible reason for the position-specific transportation is the interplay among interfacial tension, secondary flows, and mass transfer. In previous studies on the mass transfer mechanism of liquid-liquid slug flow, surface tension change by the transportation of solute has been neglected for the model simplification. A further detailed investigation by computational fluid dynamics with considering the interplay among interfacial tension, secondary flows, and mass transfer are necessary to clarify the mechanism behind the position-specific mass transfer.

## Methods

### Materials

Bromothymol blue, 1 M nitric acid, 1 M sodium hydroxide solution, dodecane, and 1 M hydrochloric acid were purchased from Fujililm Wako Pure Chemical Industry (Osaka, Japan) and used as received. Milli-Q ultra-purified water was used for all the experiments.

### Hardware

Valving system was constructed with solenoid valves (LVM105R-6G2U and LVM10R4-6G2U, SMC Corporation, Tokyo, Japan), solid-state relays (AQZ207, Panasonic), and a multifunction input/output device (USB-6001, National Instruments). The response time was below 10 ms for the solenoid valves. YSP-301 syringe pumps (YMC Co., LTD., Kyoto, Japan) and Phd Ultra syringe pumps (Harvard Apparatus, MA) were controlled by a serial command from the PC.

Tubing, tee, and elbows made of PFA were purchased from Nippon Pillar Packing Co., LTD (Osaka, Japan). Two types of tubing, 3.18 mm (1/8”) outer diameter (OD) with 2.18 mm ID, and 3 mm ODwith 2 mm ID were used. Tees and elbows had an ID of 2.1 mm. Panels with meandering channels were 3D-printed; each panel had two carved sections among 10 mm, 30 mm, and 10 mm straight parts. The first curved part was a half circle with a 5 mm inner radius. The second curved part was a quarter circle with a 5 mm inner radius. PFA tubing thickness accuracy is ±0.05 mm. To avoid the errors owing to tubing thickness, the volume of the channel was measured before the experiment. The weight of the water filling the channel was used to calculate the internal volume.

Optical sensor with a red LED light source (FS-N11MN, KEYENCE, Osaka, Japan) was employed for slug detection. A fiber couple with a spot diameter of 0.5 mm (FU-75F, KEYENCE) was placed on a sorting tee using a 3D-printed attachment. The distance between the channel branching point and the spot center was about 5 mm. The signal from the sensors was acquired by USB-6001 at 20,000 datapoints per second.

### Mass transfer experiment

Acidic sender aqueous solution contained 0.2 mM BTB, 0.5 M HNO_3_ and 1.71 M (8 wt%) ethanol; basic receiver aqueous solution contained 0.5 M NaOH and 1.71 M (8 wt%) ethanol. Ethanol was added to the sender solution to increase the solubility of BTB; it was also added to the receiver solution to ensure the same bulk liquid properties. Solutions were fed by the “hybrid control” method described in the Supplementary Information, SI1. Before exchanging the liquids, the channel was washed with acetone, water and dried with air.

BTB concentration at the outlet was determined with a V-730 (JASCO, Tokyo, Japan) UV-Vis spectrophotometer for an absorbance peak at 618 nm. The acidic sample was diluted by a basic solution of 0.5 M NaOH and 1.71 M ethanol before the measurement for adjusting the pH.

### Velocity measurement

PFA tubing with 1/8” OD and 2.18 mm ID, 1 m length was set as a straight channel. 50 cm length around the center of the channel was marked as the velocity measurement zone. Solutions were fed by the “hybrid control” method described in the Supplementary Information, SI1. Slug movement inside the measurement zone was captured by a DSC-WX500 digital camera (Sony, Tokyo, Japan). Velocities were studied at least 3 times for each experimental condition. The standard deviation of measured velocity was below 0.2% in all experiments corresponding to the camera flame rate of 60 frames per second. Before exchanging the liquids, the channel was washed with acetone, water and dried with air.

## Supplementary information

Supporting Movie 1

Supporting Movie 2

Supporting Movie 3

Supporting Movie 4

Supporting Movie 5

Supplementary Information.

## Data Availability

The datasets generated during and/or analyzed during the current study, and the software to control equipment are available from the corresponding author on reasonable request.
