# Supplementary material for: Contactless mass transfer for intra-droplet extraction
Source: Sci Rep. 2020 May 6;10:7685. doi: 10.1038/s41598-020-64520-4 (PMC7203142; doi:10.1038/s41598-020-64520-4)
Supplement: Supplementary file 6 — Supplementary Information. [file 41598_2020_64520_MOESM6_ESM.pdf]

Supplementary Information for

Contactless mass transfer intra-droplet extraction

Shusaku Asano[1,2*], Yu Takahashi[3], Taisuke Maki[3], Yosuke Muranaka[3], Nikolay Cherkasov[4] , Kazuhiro Mae[3]

[1]Institute for Materials Chemistry and Engineering, Kyushu University, 6-1, Kasuga Koen, Kasuga 816-8580, Japan
[2]Interdisciplinary Graduate School of Engineering Sciences, Kyushu University, 6-1, Kasuga Koen, Kasuga 816-8580, Japan
[3]Department of Chemical Engineering, Graduate School of Engineering, Kyoto University, Kyoto-daigaku Katsura, Nishikyo-ku, Kyoto 615-8510, Japan
[4]School of Engineering, University of Warwick, Coventry CV4 7AL, United Kingdom
*Correspondence and requests for materials should be addressed to S.A. (email: shusaku_asano@cm.kyushu-u.ac.jp)

SI1. Slug formation methods

Three different operating procedures for slug formation were tested for consistency of operation:
1) **Valve control**. All the syringe pumps are continuously running and solenoid valves open one by one when required.
2) **Pump control**. Syringe pumps run and stop one by one when required and solenoid valves are always open.
3) **Hybrid control**. Syringe pumps run and stop one by one when required and their corresponding solenoid valves open and close at the same time.

The stability of droplet formation using these three methods were examined using two different syringe pumps and various total flow rates. A low-cost YSP syringe pump (model YSP-301 & 202, YMC Co., LTD, Japan) was compared to a more expensive PHD ULTRA pump (model PHD ULTRA, Harvard Apparatus, MA). The YSP pump costs less than a quarter of Harvard Apparatus pump. Total flow rate, F_t was set to 1 mL min^{-1} or 10 mL min^{-1}. The fraction of the aqueous phase of the total flow rate (α) was 0.5. The set slug length (l) was 15 mm for two aqueous slugs and was calculated from the inner diameter (2.18 mm) of the employed PFA (perfluoroalkoxy alkane) tubing, flow rate, and frequency of the slug/pump switching. Meniscus and film at the wall were neglected assuming cylindrical droplet shape.

Table S1. Comparison of slug formation methods with changing syringe pumps and flow rate. (Aqueous fraction in total flow was $\alpha = 0.5$, and set slug length l = 15 mm for all the conditions.)

	Total flow rate F_t	
	1.0 mL min^{-1}	10 mL min^{-1}
Valve control (PHD ULTRA pump)	Works with pulsation	Works with pulsation
Pump control (PHD ULTRA pump)	**Works consistently**	Not consistent
Hybrid control (PHD ULTRA pump)	**Works consistently**	**Works consistently**
Valve control (YSP pump)	Works with pulsation	Works with pulsation
Pump control (YSP pump)	Not consistent	Not consistent
Hybrid control (YSP pump)	**Works consistently**	Not consistent

The results are summarized in **Table S1** and **Supporting Movie 1**. The valve control method worked regardless of the syringe pump model although pulsation occurred at the moment of valve opening. On the other hand, the quality of the other methods strongly depended on the syringe pump. With the YSP pumps, slug lengths were uncontrollable, especially at the higher flow rate. With the PHD ULTRA pumps, the hybrid control method worked accurately without pulsation. The pump control method was feasible at F_t = 1.0 mL min^{-1}. At 10 mL min^{-1} where the flow was moving when the time pump stopped probably due to the inertial movement so that the pump control method was suffered from the slug splitting at the tee. Therefore, the hybrid control method with the PHD ULTRA pumps was selected and used in the work due to its high flow consistency.

The problems with using low-cost syringe pumps were likely caused by low accuracy in the flow rate and slow response time. The PHD ULTRA syringe pump features ± 0.35% accuracy with ±0.05% reproducibility.[1] Low-cost syringe pumps have a much higher error value of ±1%.[2] Response times are not specified in the brochures, however, we needed about 0.1-0.2 s lead time for accurately communicate with YSP syringe pumps via ASCII commands. The valve control method, in which the pumps are always running, has inevitably made pulsation due to the pressure increase in the closing time and pressure release at valve opening. However, the valve control method can allow pumps to continuously run to avoid the problem regarding the response time delay. The response time of the solenoid valves for low-pressure fluid employed in this study was around 10 ms, which was much faster than that of the syringe pumps. Although the hybrid control with high-quality syringe pumps showed the best performance, the valve control method with low-cost syringe pumps might be a feasible choice considering the syringe pumps cost was the highest for constructing the current system. The pump control method might be the choice for applications with a low flow rate.

SI2. Using the optical sensor for the slug sorting

Figure 2 of the main manuscript shows that the optical sensor was detecting the difference between the oil and aqueous droplets. Some spikes were recorded when the meniscuses of the slugs were at the sensing point. The difference in signal likely comes from various refractive indexes of oil and water. The durations of high voltage and low voltage were not constant because of a velocity distance between alternating slugs.

We made a program that switched the two output valves. The average voltage value between the higher and the lower plateaus was set as a threshold. When the output voltage from the sensor showed a lower value than the threshold, the valves were switched. In this manner, the alternating aqueous slugs were successfully led to different outlets. Although the fiber sensor with a red light source was employed, a liquid sensor with an infrared LED light source[3] was also tested and succeeded in the detection of the slugs.

SI3. Fluid properties

Density and viscosity of 8wt% ethanol solution and dodecane were obtained from literature[4,5]. Diffusivity of BTB was estimated based on the Wilke-Chang equation.[6] It was $3.11 \cdot 10^{-10}$ m^2 s^{-1} in dodecane and $3.11 \cdot 10^{-10}$ m^2 s^{-1} in the 8wt% ethanol solution. Interfacial tensions of aqueous solutions and dodecane were measured by the pendant drop method (SI4). **Table S2** summarizes the interfacial tensions at room temperature (293K). When the oil phase was pure dodecane, pendant drops formed in a reproducible manner. At least 10 photographs were analyzed for calculating interfacial tension for each case. The measured value for dodecane and water system agreed well with the literature value[7] of 52.55 mN m^{-1}. However, when the oil phase contained BTB, a pendant drop became unstable and measurement was not reproducible.

Table S2. Interfacial tensions at room temperature

Oil phase	Aqueous phase	Interfacial tension [mN m^{-1}]
dodecane	water	52.41
dodecane	8 wt% ethanol, 0.5 M HNO_3 in water (sender solution without BTB)	33.57
dodecane	8 wt% ethanol, 0.5 M HNO_3, 0.2 mM BTB in water (sender solution)	20.84
1 mM BTB in dodecane	8 wt% ethanol, 0.5 M HNO_3 in water (receiver solution)	19-20

SI4. Pendant drop method

Quartz cell with 10 mm x 10 mm cross-section was filled with dodecane. 1/32 SUS tubing (IDEX Health & Science, U-1114) was perpendicularly dipped to dodecane and the aqueous solution was slowly fed at the flow rate of 0.01 mL/min. The movie was recorded by a digital microscope (KEYENCE, Osaka, Japan, Model: VHX-1000) till the droplet left the tip of the needle. The surface tension of two liquids, γ, were calculated from the equatorial diameter *D* and the diameter *d* at the distance *D* from the top of the drop as follows:

$$\gamma = \frac{\Delta \rho g D^2}{H}$$

$$\frac{1}{H} = \frac{B_4}{S^a} + B_3 S^3 - B_2 S^2 + B_1 S - B_0$$

$$S = d/D$$

a and B_i are empirical parameters. They are calculated according to the table in the reference.[8] **Figure S1** shows the drop with D and d.

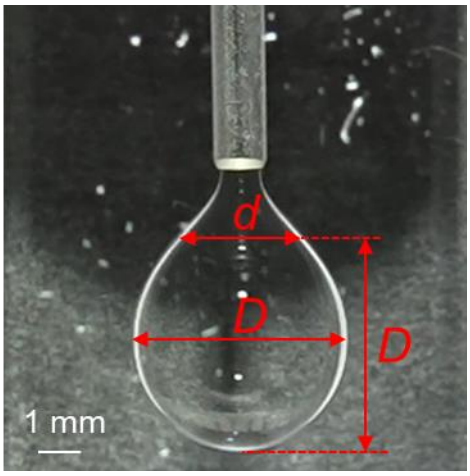

Figure S1. Pendant drop of water in dodecane before dripping.

SI5. Dimensionless parameters

A film thickness between the wall and the slugs, δ, were calculated according to the Bretherton's equation.

$$\frac{\delta}{r} = 1.34\mathrm{Ca}^{2/3}$$

r is the channel inner radius and Ca is the capillary number defined with continuous phase viscosity μ.

$$\mathrm{Ca} = \mu u_s / \gamma$$

u_s is the superficial slug velocity. A typical operation condition of 10 mL/min gives the Ca of 0.00321 and film thickness of 31.8 µm.

The Fourier number was defined as:

$$\mathrm{Fo} = \frac{\text{slug residence time on a film}}{\text{solute diffusion time from film}} = \frac{l/u_s}{\delta^2/D_S}$$

Where l is the slug length, D_S is the diffusivity of the solute, δ is the film thickness.

Typical slug length of 15 mm results in the Fo of 0.11. Fo < 1 means mass transport from film to slug is not so fast to be completed only by the molecular diffusion.

References

(1) PHD ULTRA Brochure https://www.harvardapparatus.com/.

(2) New Era Pump Systems, 2013 Catalog http://www.syringepump.com/.

(3) Cherkasov, N.; Expósito, A. J.; Bai, Y.; Rebrov, E. V. Counting Bubbles: Precision Process Control of Gas-Liquid Reactions in Flow with an Optical Inline Sensor. *React. Chem. Eng.* **2019**, *4* (1), 112–121. https://doi.org/10.1039/c8re00186c.

(4) Matsuoka, A.; Noishiki, K.; Mae, K. Experimental Study of the Contribution of Liquid Film for Liquid-Liquid Taylor Flow Mass Transfer in a Microchannel. *Chem. Eng. Sci.* **2016**, *155*, 306–313. https://doi.org/10.1016/j.ces.2016.08.021.

(5) Khattab, I. S.; Bandarkar, F.; Fakhree, M. A. A.; Jouyban, A. Density, Viscosity, and Surface Tension of Water+ethanol Mixtures from 293 to 323K. *Korean J. Chem. Eng.* **2012**, *29* (6), 812–817. https://doi.org/10.1007/s11814-011-0239-6.

(6) Bird, R. B.; Stewart, W. E.; Lightfoot, E. N. *Transport Phonomena*, Revised 2n.; John Wiley & Sons, Inc., 2006.

(7) Zeppieri, S.; Rodríguez, J.; López De Ramos, A. L. Interfacial Tension of Alkane + Water Systems. *J. Chem. Eng. Data* **2001**, *46* (5), 1086–1088. https://doi.org/10.1021/je000245r.

(8) Hubbard, A. T. *Encyclopedia of Surface and Colloid Science, Third Edition*; Somasundaran, P., Ed.; CRC Press, 2002. https://doi.org/10.1081/E-ESCS3.
